# Triglyceride–glucose index as a metabolic risk marker for adverse outcomes after coronary artery bypass grafting: a systematic review and meta-analysis

**DOI:** 10.3389/fendo.2026.1909829

**Published:** 2026-09-11

**Authors:** Yuhan Zhang, Yifan Zeng, Yirui Ning, Huiyu Wang, Xinwu Yang, Xiaobo Liao, Zhiyuan Zhang

**Affiliations:** 1 Department of Cardiovascular Surgery, The Second Xiangya Hospital of Central South University, Changsha, Hunan, China; 2 Xiangya School of Medicine, Central South University, Changsha, Hunan, China; 3 The Second Xiangya Hospital of Central South University, Changsha, Hunan, China; 4 The First Clinical Medical College, Shanxi Medical University, Taiyuan, Shanxi, China; 5 Hunan Provincial Clinical Research Center for Cardiovascular Surgery, Changsha, Hunan, China

**Keywords:** coronary artery bypass grafting, insulin resistance, major adverse cardiovascular events, meta-analysis, triglyceride–glucose index

## Abstract

**Aims:**

To evaluate the prognostic value of the triglyceride–glucose index (TyG), a surrogate marker of insulin resistance, for adverse cardiovascular outcomes in patients undergoing coronary artery bypass grafting (CABG).

**Materials and methods:**

This systematic review and meta-analysis was conducted in accordance with the Preferred Reporting Items for Systematic Reviews and Meta-Analyses (PRISMA) guidelines. Studies evaluating the association between the TyG index and postoperative outcomes after CABG were retrieved from four databases as of Jul 10, 2026. Multivariable-adjusted hazard ratios (HRs) with 95% confidence intervals (CIs) were used as the primary effect estimates for major adverse cardiovascular events (MACE), with separate analyses conducted for each 1-unit increase in the TyG index and for TyG index tertiles. Crude odds ratios (ORs) comparing the higher TyG group with the lower TyG group were estimated as a supplementary event-based analysis.

**Results:**

Six retrospective cohort reports, comprising 5,978 participants and four independent cohort families, were included, with two being multi-center studies. Each 1-unit increase in the TyG index was associated with a 62.4% higher hazard of MACE (HR: 1.624, 95% CI: 1.349-1.955). Compared with the lowest tertile group, the highest tertile group demonstrated a statistically higher hazard of MACE (HR = 2.093, 95% CI: 1.635-2.680). A higher TyG index was associated with higher odds of MACE (OR: 2.59, 95% CI: 1.97-3.40). In addition, a higher TyG index was associated with an elevated risk of all-cause mortality, nonfatal stroke, and nonfatal myocardial infarction, but was not associated with revascularization.

**Conclusion:**

The TyG index may serve as an accessible supplementary metabolic risk marker following CABG. Further prospective validation is required before it is routinely applied to individual risk prediction.

**Systematic Review Registration:**

https://www.crd.york.ac.uk/PROSPERO/, identifier CRD420251158075

## 1 Introduction

Coronary artery disease (CAD) remains one of the leading contributors to mortality, disability, and healthcare resource depletion worldwide (1). For patients with severe multivessel disease, coronary artery bypass grafting (CABG) effectively restores the myocardial blood supply in CAD patients. It remains the preferred revascularization strategy for treating CAD (2). Postoperative complications following CABG surgery may result in substantial morbidity and mortality. Despite advances in surgical techniques that have enhanced the efficacy and safety of CABG, the incidence of major adverse cardiovascular events (MACE) in postoperative patients is still consistently elevated (3).

Diabetes patients are at greater risk of cardiovascular disease. Insulin resistance (IR) is a critical risk factor for cardiovascular metabolic disorders and a hallmark feature of type 2 diabetes mellitus (T2DM). It accelerates atherosclerosis progression through proinflammatory and prothrombotic actions, playing a pivotal role in the pathogenesis of CAD (4). The triglyceride–glucose index (TyG), a reliable surrogate marker for IR, is calculated as the product of triglyceride and glucose concentrations. This noninsulin-based marker of IR offers greater cost-effectiveness and convenience than conventional approaches, with results that are highly consistent with those from hyperinsulinemic–euglycemic clamp studies (5, 6). The TyG index has been proven to be a valuable tool in the comprehensive management of patients with CAD, and its correlation with CAD has been validated in multiple studies. Xia and colleagues confirmed that the incidence of atherosclerotic cardiovascular disease (ASCVD) increased with increasing TyG index levels (7). Another meta-analysis by Liang et al. indicated that patients with the highest TyG index exhibited a heightened risk of CAD compared with those with the lowest index, developing more coronary artery stenosis, plaque progression, and wider vascular involvement (8). Some studies have also reported an independent link between an elevated TyG index and MACE, including in patients with CAD (9) and those undergoing percutaneous coronary intervention (PCI) (10–13). Recent studies have suggested that a higher TyG is associated with an increased risk of MACE after CABG. Chen et al. reported that the TyG index was an essential predictor of adverse cardiovascular outcomes in patients with T2DM after CABG (14). The value of the TyG index in predicting adverse cardiovascular events following CABG in non-diabetic patients has also been documented (15). Sun and colleagues demonstrated that among triglyceride-to-high-density lipoprotein cholesterol (TG/HDL-C) and the metabolic score for IR (METS-IR), TyG exhibited optimal stability in predicting outcomes for CABG patients (16). Ruan et al. demonstrated that the TyG index is a reliable prognostic indicator for MACE after CABG in patients with ischemic cardiomyopathy and heart failure with preserved ejection fraction (HFpEF), serving as a valuable complement to traditional cardiovascular risk factors by providing metabolic-related insights (17). Nevertheless, no meta-analysis has yet investigated the predictive value of the TyG index for MACE following CABG. Therefore, this research systematically examined the relationship between the TyG index and the incidence of MACE in the post-CABG population, aiming to evaluate the potential prognostic role of the TyG index in CAD patients undergoing CABG.

## 2 Methods

This systematic review and meta-analysis strictly adhered to the Preferred Reporting Items for Systematic Reviews and Meta-Analyses (PRISMA) statement (18). This protocol has been registered in the International Prospective Register of Systematic Reviews (PROSPERO) on Sep 29, 2025 (CRD420251158075; https://www.crd.york.ac.uk/PROSPERO/view/CRD420251158075).

### 2.1 Literature search

Two authors (YHZ and YRN) conducted the literature search by combining Medical Subject Headings (MeSH) with free-text terms across four major databases (PubMed, Web of Science, Embase, and the Cochrane Library). The search scope covered the period from the inception of each database up to Jul 10, 2026. The keyword search terms used were as follows: (1) TyG index: ‘TyG index’ OR ‘triglyceride glucose index’ OR ‘triglyceride-glucose index’ OR ‘triglyceride and glucose index’. (2) For CABG: ‘Coronary Artery Bypass’ OR ‘Coronary Artery Bypass, Off-Pump’ OR ‘Internal Mammary-Coronary Artery Anastomosis’. Keywords were subsequently modified according to the specific constraints of each database. The detailed search strategies for each database are delineated in **Supplementary Table 1**. In addition, the reference lists of all included studies were manually verified to identify potentially overlooked relevant research.

### 2.2 Study selection and eligibility criteria

All included studies were in accordance with the population, intervention, or exposure, comparison, outcomes, and study design (PICOS) framework: (1) Population: adult patients undergoing CABG. (2) Intervention or exposure: TyG index as a predictor of clinical outcomes. (3) Outcomes: At least one relevant postoperative clinical outcome, including MACE, all-cause mortality, cardiovascular mortality, myocardial infarction, stroke, revascularization, graft dysfunction, or heart failure. The included studies had to satisfy all the components of the aforementioned PICOS framework. Research was excluded from the meta-analysis if it exhibited any of the following characteristics: (1) Concurrent procedures such as valve surgery, surgical ablation, congenital heart disease surgery, or PCI. (2) Non-CABG populations or mixed populations without separately extractable CABG data. (3) Missing data on postoperative outcomes relevant to this review. (4) Limited to patients with specific severe cardiac disease. (5) Repeated or overlapping data with a more comprehensive study. (6) Not a cohort study. (7) Non-English languages. Studies with potential overlap could not be included in the same meta-analysis.

### 2.3 Data extraction

Two researchers (YHZ and YRN) collaborated to extract and verify the data utilizing a standardized Excel spreadsheet and consulted a third researcher (HYW) to resolve any discrepancies. The following characteristics were extracted: (1) First author information and publication year. (2) Study design type and follow-up duration. (3) Participant characteristics, including sample size, age, sex distribution, and diabetes status. (4) Methods used to analyze and classify the TyG index, including reported cut-off values. (5) Components of the MACE outcome. (6) Covariates included in the multivariable-adjusted models. In addition, information regarding potential cohort overlap was extracted, including participating centers, recruitment periods, patient populations, and inclusion and exclusion criteria. A comparative analysis of the studies was conducted to assess the possibility of duplicate or overlapping participants. The main research characteristics are summarized in **Table 1**, while detailed information for identifying potential cohort overlaps is included in **Appendix 1**. The extracted outcome data presented in **Appendix 2** included: (1) Number of participants and events in each TyG category across each study, which were used to calculate odds ratios (ORs). (2) Multivariably adjusted hazard ratios (HRs) per one-unit increase in the TyG index and corresponding 95% confidence intervals (CIs). (3) Multivariably adjusted HR across TyG tertiles and corresponding 95% CIs. (4) Prognostic/predictive indices. (5) Incremental predictive value, including continuous net reclassification improvement (NRI) and integrated discrimination improvement (IDI).

**Table 1:** Characteristics of the included studies.

Author (Year)	Study design	Patients, n (M/F)	Age (years)	Follow-up	TyG analysis	Definition of MACE	Diabetes status	Patient population	Categorical TyG exposure definition
Chen 2022 (14)	retrospective cohort, single-center	1578 (1116/462)	62.9 ± 8.00	24 months	continuous and categorical	①②③④	T2DM	adults with T2DM undergoing isolated OPCABG	Study-defined higher vs. lower groups; threshold NR
Zhang 2022 (23)	retrospective cohort, single-center	386 (276/110)	66.0 ± 8.89	Maximum: 60 months	categorical^*^	①②③⑤⑥	T2DM	adults with T2DM after isolated CABG	Median cut-off: 9.17
Wu 2023a (21)	retrospective cohort, single-center	1013 (709/304)	62.7 ± 8.30	69 months, median (IQR: 58–76)	continuous and categorical	①②③⑤	Not restricted to T2DM	adults undergoing first-time isolated CABG	Study-defined higher vs. lower groups; threshold NR
Wu 2023b (22)	retrospective cohort, multi-center	1225 (857/368)	62.8 ± 8.18	69 months, median (IQR: 56–76)	continuous and categorical	①②③⑤	Not restricted to T2DM	adults undergoing first-time isolated CABG	ROC-derived cut-off: >8.87 vs ≤8.87
Sun 2025 (16)	retrospective cohort, multi-center	1472 (1034/438)	62.9 ± 8.13	85 months, median (IQR: 66–96)	continuous and categorical	①②③⑤	Not restricted to T2DM	adults undergoing CABG	Tertiles; numerical boundaries NR
Wang 2026 (24)	retrospective, single-center cohort	304 (226/78)	63.0 ± 8.2	44 months, median (IQR: 31–62)	continuous and categorical	①③⑤⑥⑦	Not restricted to T2DM	adults undergoing CABG	Cut-off: 8.65

The numbered symbols indicate the following components of MACE: ① all-cause mortality; ② nonfatal myocardial infarction; ③ nonfatal stroke; ④ graft failure; ⑤ coronary artery revascularization; ⑥ heart failure; and ⑦ severe arrhythmias.

^*^ The categorical comparison of Zhang 2022, defined using a median TyG cut-off of 9.17, was retained in the high-versus-low meta-analysis. The continuous HR was not quantitatively pooled because the corresponding exposure increment was unspecified, and the underlying regression model was reported inconsistently.

Categorical TyG exposure definition refers to the method used to define exposure groups for categorical analyses. It should be distinguished from the ROC-derived optimal cut-off used for outcome discrimination.

The TyG index was calculated in all included studies using the following formula: ln [fasting TG (mg/dL) × FPG (mg/dL)/2].

CABG, coronary artery bypass grafting; F, female; FPG, fasting plasma glucose; HR, hazard ratio; IQR, interquartile range; ln, natural logarithm; M, male; MACE, major adverse cardiovascular events; MI, myocardial infarction; NA, not applicable; NR, not reported; OPCABG, off-pump coronary artery bypass grafting; ROC, receiver operating characteristic; SD, standard deviation; T2DM, type 2 diabetes mellitus; TG, triglyceride; TyG, triglyceride–glucose index.

### 2.4 Quality evaluation

The quality of each study was independently assessed by two researchers (HYW and XWY) through the Newcastle–Ottawa Scale (NOS) (19). The scale scores studies from 0 to 9 points, evaluating cohort study quality based on three criteria: study selection, comparability of groups, and assessment of outcomes. Study quality was categorized as high (≥ 8 points), moderate (5–7 points), or low (< 5 points).

### 2.5 Statistical analysis

Meta-analyses were conducted to investigate the association between the TyG index and post-CABG MACE risk. Multivariably adjusted HRs were regarded as the primary effect estimates. Where multiple adjustment models were presented within a study, estimates from the most comprehensively adjusted model were selected. HRs were pooled separately by TyG index category. For continuous analyses, HRs per 1-unit increase in the TyG index were combined. For categorical analyses, HRs comparing the highest tertile (T3) with the lowest tertile (T1), and HRs comparing the middle tertile (T2) with T1 were pooled separately, with T1 serving as the reference group. Crude ORs and 95% CIs were calculated from cumulative event counts in the higher and lower TyG groups as defined by each study. Since ORs did not account for covariate adjustment or varying follow-up durations, they were considered a supplementary event-based analysis and not regarded as comparable to multivariable-adjusted survival estimates. All included HRs were obtained from multivariate Cox proportional hazards regression models. The covariates are detailed in **Appendix 3**. For studies reporting HRs with 95% CIs but without standard errors (SEs), the corresponding SEs were derived using the normal (Wald) approximation and the formula SE[ln(HR)] = [ln(upper CI limit) − ln(lower CI limit)]/(2 × 1.96). When the CI is asymmetrically distributed around the published ln(HR) value, retain the published HR value and use the formula to approximate the SE. Consequently, the forest plot displayed the Wald CI reconstructed from the derived SE.

τ² and the *P*-value of the Q test were used to estimate the degree of heterogeneity (20). A Q test *P* value less than 0.10 was considered indicative of notable heterogeneity. I² values of approximately 25%, 50%, and 75% are interpreted as indicating low, moderate, and high heterogeneity, respectively. Due to heterogeneity across studies, encompassing diabetes status, duration of follow-up, components of MACE, and the limited number of studies, all meta-analyses employed a random-effects model. Exploratory subgroup analyses were conducted to assess significant heterogeneity. Leave-one-out sensitivity analysis was conducted by sequentially excluding individual studies to verify the robustness of the pooled estimates and to identify potential sources of heterogeneity. In addition, a sensitivity analysis based on the MACE definitions was performed. As fewer than 10 studies were included in each meta-analysis, no funnel plots were reported, nor were formal tests for funnel plot asymmetry carried out. Forest plots and other visualizations were uniformly generated through the Online Meta platform.

## 3 Results

### 3.1 Study inclusion and basic characteristics

**Figure 1:**
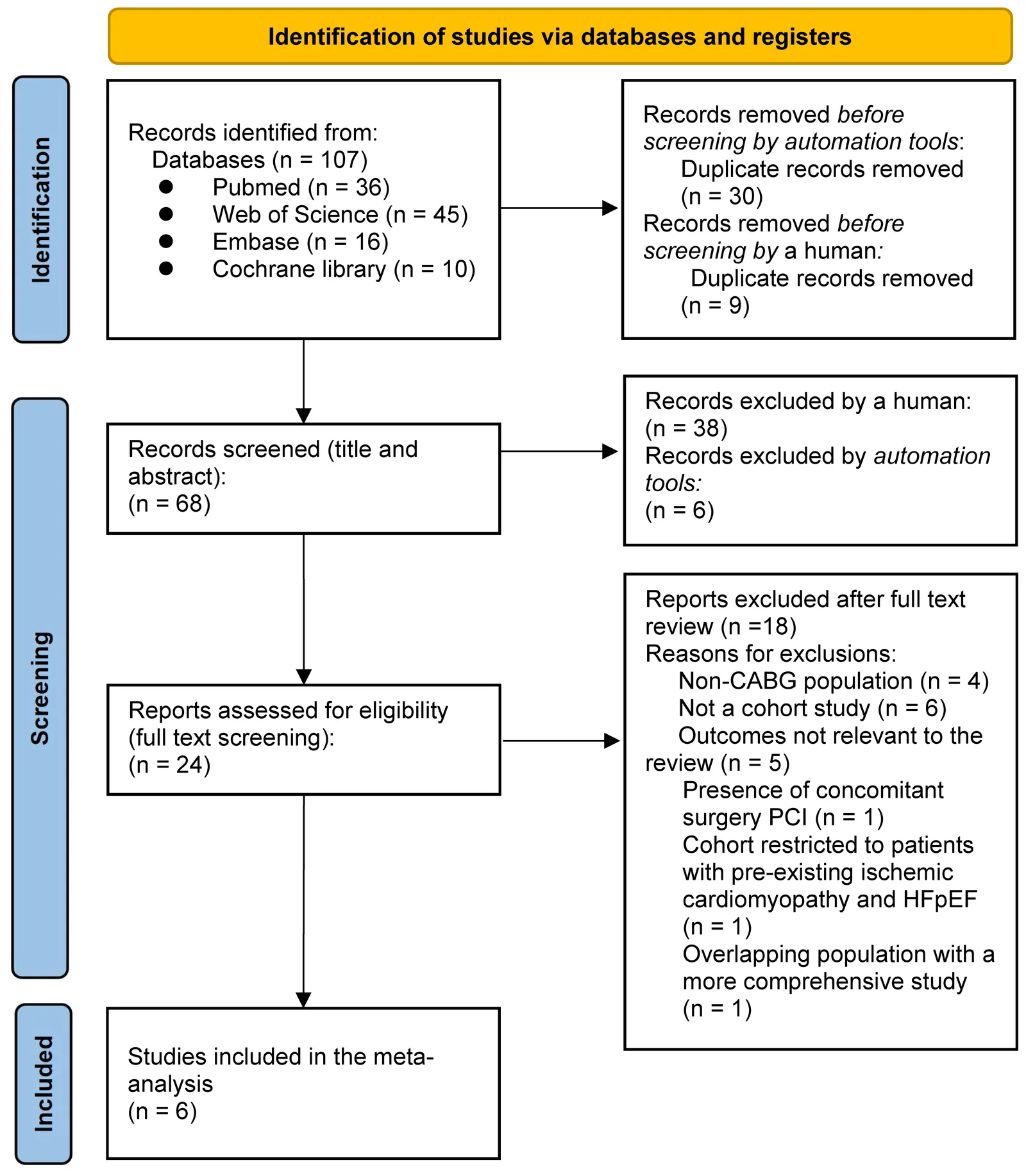
PRISMA flow diagram. CABG, coronary artery bypass grafting; HFpEF, heart failure with preserved ejection fraction; PCI, percutaneous coronary intervention; PRISMA, Preferred Reporting Items for Systematic Reviews and Meta-Analyses. Adapted from the PRISMA 2020 flow diagram template, Page et al. (18), available at https://www.prisma-statement.org/prisma-2020-flow-diagram, under the Creative Commons Attribution 4.0 International license (https://creativecommons.org/licenses/by/4.0/). The template was modified to present the study selection process for this review.

The detailed process for study selection and exclusion is shown in **Figure 1**. A total of 107 potentially relevant articles were retrieved from four databases: PubMed (n = 36), Web of Science (n = 45), Embase (n = 16), and Cochrane Library (n = 10). After 39 duplicate records were removed *via* EndNote software and manual screening, 68 articles were included in the title and abstract screening phase. Of these, 44 were excluded for not aligning with the research focus. Following the full-text evaluation of the remaining 24 studies, 18 were eliminated: 4 included non-CABG populations; 6 were not cohort studies; and 5 reported outcomes that were not relevant to the review; 1 compared concurrent PCI; 1 included only patients with pre-existing ischemic cardiomyopathy and HFpEF; and the participant cohort in another study overlapped with more comprehensive studies. Ultimately, 6 cohort studies were selected for the meta-analysis. No randomized controlled trials (RCTs) were included.

The baseline characteristics of the enrolled studies are summarized in **Table 1**. A total of 5,978 patients were included across all 6 publications, representing four independent cohort families (14, 16, 21–24). Notably, Wu 2023a, Wu 2023b, and Sun 2025 are considered to represent extremely overlapping Shandong CABG cohort studies. Four of these were single-center studies, and two were multi-center studies, with sample sizes ranging from 304 to 1,578 patients and average ages ranging from 62.7 to 66.0 years. The reported follow-up periods spanned 24 to 85 months. Two studies included only patients with T2DM, while the remaining four did not restrict enrollment by diabetes status. The TyG index was analyzed as a continuous variable, a categorical variable, or both in the included studies, calculated using the same formula: ln[fasting triglyceride (mg/dL) × fasting plasma glucose (mg/dL)/2]. Three studies reported cut-off values for TyG, ranging from 8.65 to 9.17. MACE was measured in all six studies, albeit with varying component compositions.

### 3.2 Risk of bias assessment

The methodological quality of the included studies was assessed using the NOS, with detailed scoring results presented in **Appendix 4**. All six included studies achieved NOS scores of 5–7 points, indicating moderate methodological quality. A majority of studies adequately represented the exposed cohort, controlled for key confounding factors such as body mass index (BMI) and smoking status, and provided clear estimates of outcomes. Despite slight limitations, such as incomplete follow-up, the overall risk of bias was deemed acceptable.

### 3.3 Association between TyG index and clinical outcomes

#### 3.3.1 Primary outcome: MACE

##### 3.3.1.1 Primary time-to-event analyses: adjusted HRs

**Figure 2:**
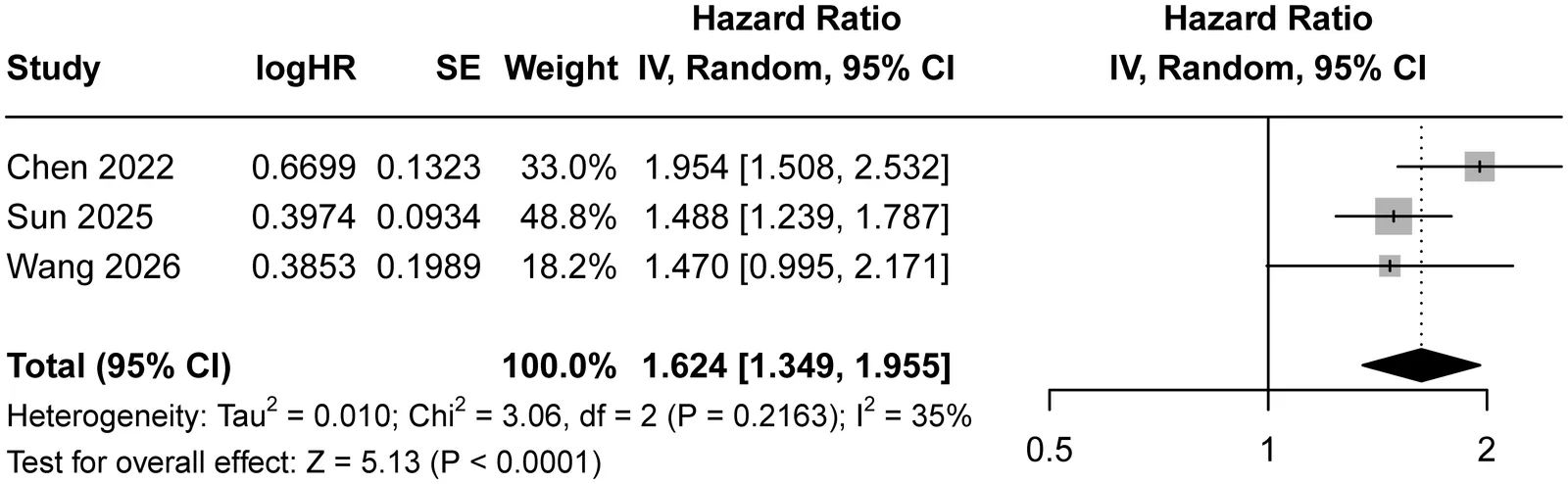
Forest plot of the association between each 1-unit increase in the TyG index and the hazard of MACE. CI, confidence interval; HR, hazard ratio; IV, inverse variance; logHR, logarithm of the hazard ratio; MACE, major adverse cardiovascular events; SE, standard error; TyG, triglyceride–glucose index.

Three independent cohorts were included in the primary time-to-event analysis. To assess the effect of each one-unit increment in the TyG index on MACE, a meta-analysis using a random-effects model was conducted based on the HRs reported in individual studies (**Figure 2**). The HR was 1.624 (95% CI: 1.349–1.955; Z = 5.13; *P* < 0.001), suggesting a higher hazard of MACE for each 1-unit increase in the TyG index. Low to moderate heterogeneity was observed (*P* = 0.216, I² = 35%).

To further assess the robustness of the findings, a sensitivity analysis using the leave-one-out approach was conducted (**Appendix 5**). The results revealed that the HR values remained consistent between 1.485 and 1.762, with all corresponding 95% CIs above 1, supporting that the direction and statistical significance of the association were not materially altered. Notably, the exclusion of Chen et al. reduced I² from 35% to 0%, while the pooled analysis remained significant (HR: 1.485, 95% CI: 1.258–1.752).

Two independent cohorts additionally reported adjusted HRs stratified by TyG tertiles (**Appendix 6**). Compared with the lowest tertile (T1), the highest tertile (T3) was significantly associated with an elevated hazard of MACE (HR = 2.093, 95% CI: 1.635–2.680; I² = 0%; *P* < 0.001), whereas the relationship for the middle tertile (T2) did not attain statistical significance (HR = 1.370, 95% CI: 0.952–1.972; I² = 46%; *P* = 0.09).

##### 3.3.1.2 Supportive event-based analysis: OR for high vs. low TyG index

**Figure 3:**
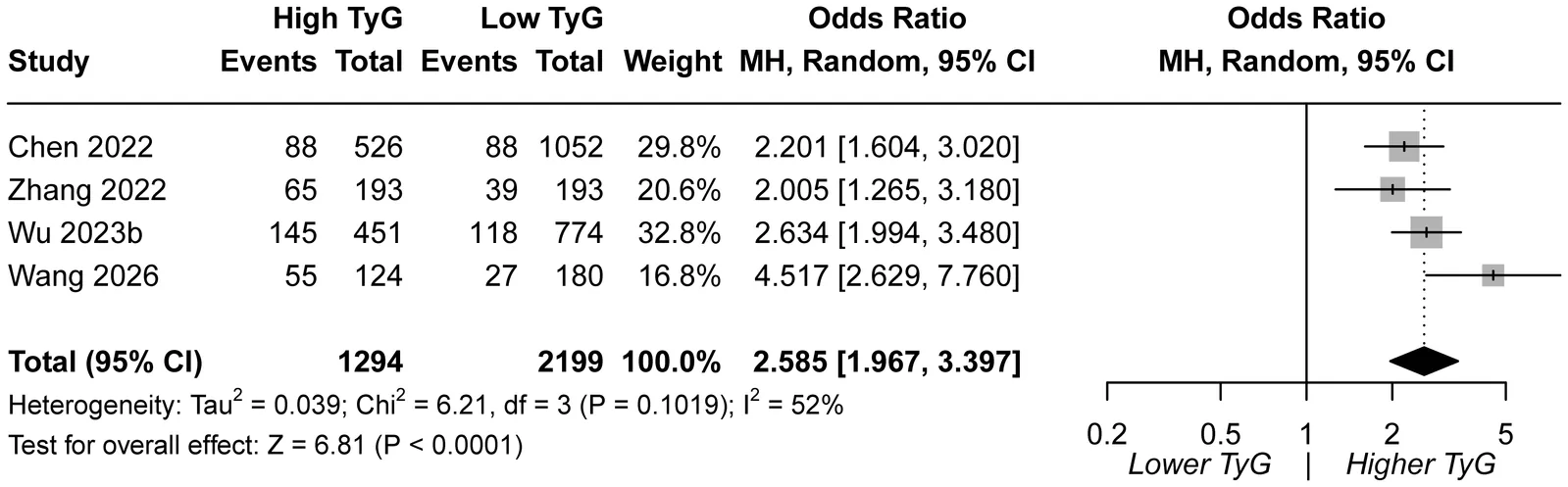
Forest plot of the association between higher versus lower TyG index groups and MACE. CI, confidence interval; MACE, major adverse cardiovascular events; MH, Mantel–Haenszel; OR, odds ratio; TyG, triglyceride–glucose index.

A meta-analysis of four studies using a random effects model yielded the findings presented in **Figure 3**. The pooled analysis demonstrated that individuals with higher TyG indices had a higher odds of MACE, with an OR of 2.585 (95% CI: 1.967–3.397; Z = 6.81; *P* < 0.001).

Moderate between-study heterogeneity was observed (I² = 52%, *P* = 0.102). To investigate potential sources of heterogeneity and assess the robustness of the results, sensitivity analyses were conducted employing a leave-one-out approach (**Appendix 7**). After each study was sequentially excluded, the ORs ranged from 2.356 to 2.793, with all 95% CIs failing to include 1. Notably, excluding the study by Wang et al. (2026) reduced heterogeneity to I² = 0%, suggesting that this study may have contributed substantially to the observed heterogeneity.

To investigate potential sources of heterogeneity, exploratory subgroup analyses were conducted by diabetes status and by whether heart failure was included in the composite definition of MACE (**Appendix 8**). In cohorts restricted to patients with T2DM, the pooled OR was 2.136 (95% CI: 1.646–2.773; I² = 0%), whereas in the overall CABG population, the corresponding OR was 3.275 (95% CI: 1.950–5.500; I² = 67%). Nevertheless, the difference between subgroups was not statistically significant (*P* = 0.149). Similarly, the pooled OR was 2.436 (95% CI: 1.976–3.002; I² = 0%) when heart failure was excluded from the MACE definition, whereas 2.971 (95% CI: 1.341–6.582; I² = 80%) when heart failure was included. None of the differences between subgroups were statistically significant (*P* = 0.636).

Given the variations in definitions of MACE across studies, a definition-based sensitivity analysis was conducted, including studies in which the composite endpoints comprised the four shared core components: all-cause mortality, nonfatal myocardial infarction, nonfatal stroke, and coronary revascularization (**Appendix 9**). The association remained consistent (OR = 2.449, 95% CI: 1.930–3.108; I² = 0%; *P* < 0.001).

#### 3.3.2 Secondary outcome: individual components of MACE

Composite MACE definitions varied across studies. All-cause mortality and nonfatal stroke were the most consistently reported components. In contrast, the inclusion of nonfatal myocardial infarction, repeat revascularization, graft failure, heart failure, and severe arrhythmias differs by study. Therefore, the following component analysis is considered a secondary comparison of events based on the current cumulative count.

##### 3.3.2.1 All-cause mortality

**Figure 4:**
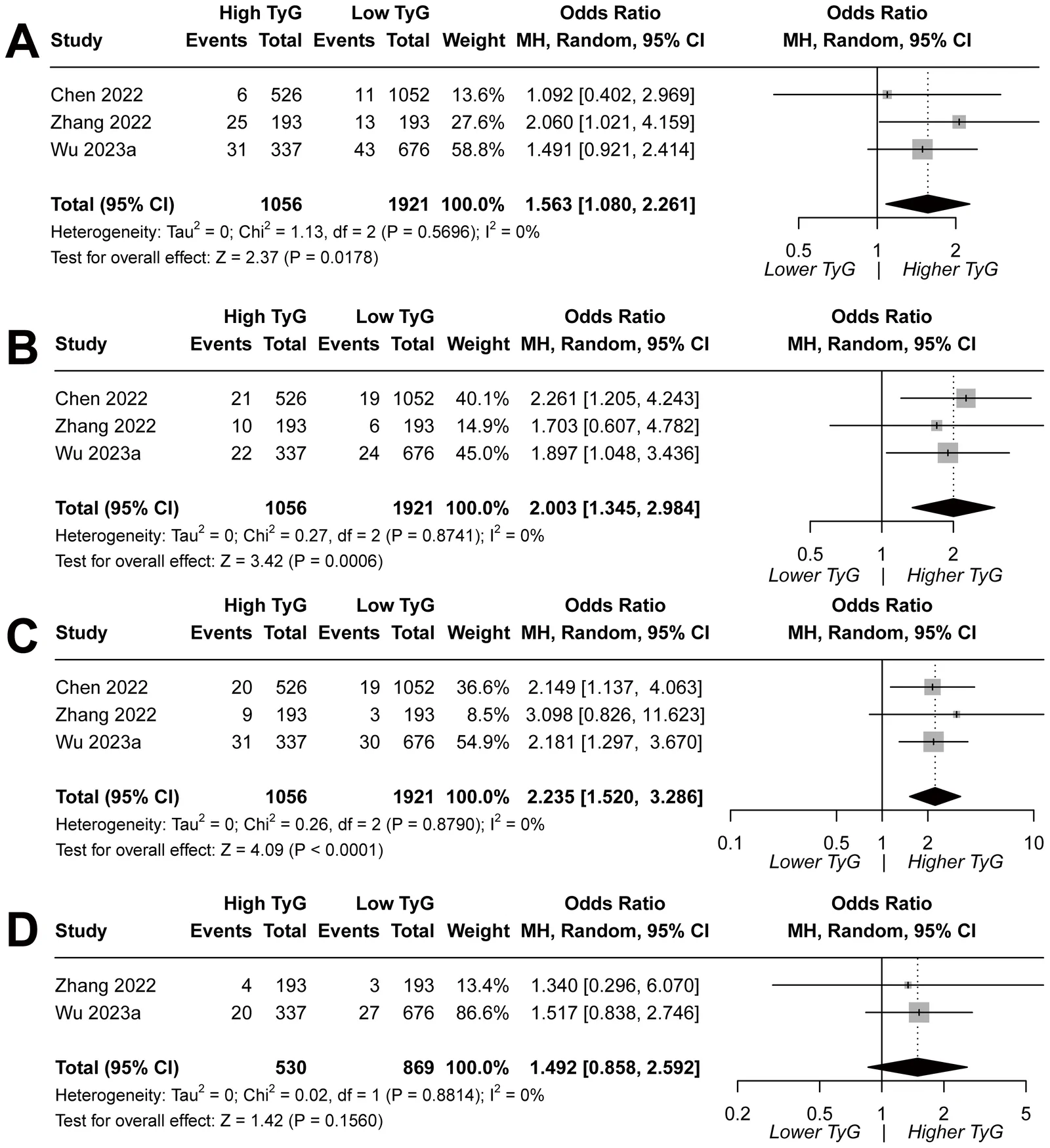
Forest plots of the association between the TyG index and individual components of MACE. **(A)** All-cause mortality. **(B)** Nonfatal stroke. **(C)** Nonfatal myocardial infarction. **(D)** Revascularization. CI, confidence interval; MACE, major adverse cardiovascular events; MH, Mantel–Haenszel; OR, odds ratio; TyG, triglyceride–glucose index.

A random effects meta-analysis compared all-cause mortality between the low- and high-TyG index groups (**Figure 4A**). The OR was 1.563 (95% CI: 1.080–2.261; Z = 2.37; *P* = 0.018), with no observed heterogeneity (*P* = 0.570, I² = 0%).

##### 3.3.2.2 Nonfatal stroke

As shown in **Figure 4B**, compared with individuals with lower TyG indices, those with higher TyG indices presented elevated odds of nonfatal stroke. The OR was 2.003 (95% CI: 1.345–2.984; Z = 3.42; *P* < 0.001), with no detected heterogeneity in the included studies (*P* = 0.874, I² = 0%).

##### 3.3.2.3 Nonfatal myocardial infarction

Three studies compared myocardial infarction between the low- and high-TyG index groups (**Figure 4C**). The meta-analysis revealed an association between an elevated TyG index and higher odds of myocardial infarction, with an OR of 2.235 (95% CI: 1.520–3.286; Z = 4.09; *P* < 0.001). No heterogeneity was found (*P* = 0.879, I² = 0%).

##### 3.3.2.4 Revascularization

In the meta-analysis of revascularization events (**Figure 4D**), the OR was 1.492 (95% CI: 0.858–2.592; Z = 1.42; *P* = 0.156), indicating no statistically significant difference between groups. Similarly, no heterogeneity was observed (*P* = 0.881, I² = 0%).

#### 3.3.3 Exploratory prognostic discrimination

Two studies reported data on the discriminatory power of the TyG index in predicting postoperative MACE (**Table 2**). When the TyG threshold was 8.90, Sun et al. reported an area under the curve (AUC) of 0.593 (95% CI: 0.557–0.629), with a sensitivity of 0.509 and a specificity of 0.681. Wang et al. reported an AUC of 0.624 (95% CI 0.554–0.694), a sensitivity of 0.671, and a specificity of 0.689 at a threshold of 8.65.

**Table 2:** Prognostic discrimination of study-specific TyG index cut-offs for MACE.

Study	TyG cut-off	Higher TyG, n (MACE/non-MACE)	Lower TyG, n (MACE/non-MACE)	Sensitivity	Specificity	AUC (95% CI)
Sun 2025	8.9	171/362	165/774	0.509	0.681	0.593 (0.557–0.629)
Wang 2026	8.65	55/69	27/153	0.671	0.689	0.624 (0.554–0.694)

The higher TyG category was treated as the positive classification, and the occurrence of MACE was treated as the positive outcome. Sensitivity was calculated as the number of patients in the higher TyG group who experienced MACE divided by the total number of patients who experienced MACE. Specificity was calculated as the number of patients in the lower TyG group who did not experience MACE divided by the total number of patients who did not experience MACE.

AUC, area under the curve; CI, confidence interval; MACE, major adverse cardiovascular events; TyG, triglyceride-glucose index.

#### 3.3.4 Incremental predictive value

Two independent studies reported the incremental prognostic performance of incorporating the TyG index into specific baseline models (**Table 3**). In the Wu 2023b study, after including TyG, the C-statistic increased from 0.658 (95% CI: 0.625–0.691) to 0.684 (95% CI: 0.651–0.717), corresponding to a continuous NRI of 0.299 (95% CI: 0.165–0.434) and an IDI value of 0.014 (95% CI: 0.006–0.022). In the study by Wang et al., the C-statistic for the reference model was 0.684 (95% CI: 0.592–0.717), which decreased to 0.679 (95% CI: 0.592–0.717) after integrating TyG, while the corresponding continuous NRI and IDI values were 0.315 (95% CI: 0.204–0.421) and 0.266 (95% CI: 0.163–0.369), respectively.

**Table 3:** Incremental prognostic value of adding the TyG index to study-specific reference models.

Study	Reference model	Extended model	Reference C-statistic (95% CI)	Extended C-statistic (95% CI)	Change in C-statistic	Continuous NRI (95% CI)	IDI (95% CI)
Wu 2023b	Model 3 excluding TyG and SUA	Reference model + TyG	0.658 (0.625-0.691)	0.684 (0.651-0.717)	0.026	0.299 (0.165-0.434)	0.014 (0.006-0.022)
Wang 2026	Model 3 excluding TyG and EAT	Reference model + TyG	0.684 (0.592-0.717)	0.679 (0.592-0.717)	−0.005	0.315 (0.204-0.421)	0.266 (0.163-0.369)

C-statistics, continuous NRI, and IDI were extracted directly from the original publications. Changes in the C-statistic were calculated as the extended-model value minus the reference-model value. Continuous NRI and IDI are presented on their original additive scales, with 0 representing no incremental predictive improvement. The covariates included in each study-specific Model 3 are detailed in Additional file 1 (**Appendix 3**).

CI, confidence interval; TyG, triglyceride-glucose index; SUA, serum uric acid; EAT, epicardial adipose tissue; NRI, net reclassification improvement; IDI, integrated discrimination improvement.

## 4 Discussion

### 4.1 Major findings

To our knowledge, this study is the first meta-analysis to examine the association between the TyG index and clinical outcomes following CABG. Based on adjusted HR values, the primary time-to-event analysis showed a positive association between the TyG index and adverse cardiovascular outcomes after CABG. Each 1-unit increase in the TyG index was associated with a 62.4% higher hazard of MACE. The supportive event-based analysis, OR for high vs. low TyG index, yielded a unifying direction of association. The secondary analysis of MACE components revealed that a heightened TyG index was linked to greater all-cause mortality, nonfatal stroke, and nonfatal myocardial infarction. These findings are consistent with previous observations in populations with CAD (7, 8, 25). Crucially, this study has extended the observations to the CABG cohort field, where existing evidence remains relatively scarce. Overall, this meta-analysis suggests that the TyG index is a promising metabolic marker for predicting high-risk MACE events following CABG.

The associations observed in the above findings are underpinned by sophisticated pathophysiological mechanisms. The TyG index represents dysregulation of fasting blood glucose and triglyceride metabolism and has been extensively used as a surrogate marker for IR (26). IR promotes endothelial dysfunction, oxidative stress, vascular inflammation, platelet activation, and an imbalance between coagulation and fibrinolysis. The pathological processes may compromise coronary microcirculation and myocardial reperfusion, while simultaneously accelerating the progression of atherosclerosis and contributing to plaque instability and rupture, thereby elevating myocardial infarction, stroke, and death (27–29). These interrelated pathways offer a plausible explanation for the association between higher TyG indices and myocardial infarction, stroke, and mortality following CABG. Nevertheless, as an intermediate metabolic marker, its prognostic relevance may also partially mirror the distinct effects of hyperglycemia and hypertriglyceridemia.

In the time-to-event analysis of HRs per 1-unit increase in the TyG index, low-to-moderate heterogeneity was observed. Through the leave-one-out method, the overall association remained statistically robust. After excluding Chen 2022, heterogeneity decreased to 0, possibly contributing to the inclusion of only T2DM individuals and relatively short follow-up. Nevertheless, owing to the limited studies, subgroup analyses were not systematically discussed. Zhang et al. also reported continuous HR. Nevertheless, the corresponding TyG increment was not explicitly specified, and there were inconsistencies in the statistical model descriptions in the original article. Therefore, the continuous estimates from this study were excluded from the meta-analysis to avoid assuming comparability with other studies. Notably, the clearly defined results comparing high and low values, based on the median of 9.17, were still included in the event-based OR analysis. The tertile-based analysis yielded limited evidence of a continuous effect, as patients in T3 had a higher incidence of MACE than those in T1. Nevertheless, the contrast between T2 and T1 was not statistically significant, and only two studies were eligible for the analysis. Therefore, the dose-response relationship could not be confirmed.

In event-based analyses, moderate heterogeneity was reported, whilst the leave-one-out sensitivity analysis did not alter the robustness of the overall association. Wang et al. contributed notably to the observed heterogeneity, possibly due to a composite endpoint that incorporates heart failure and severe arrhythmias but excludes myocardial infarction, whereas the endpoint definitions adopted in other studies more commonly encompassed myocardial infarction. Though correlation persisted in the definition-restricted sensitivity analysis, it ought to be considered supportive rather than conclusive evidence, given that only two studies are involved. No statistically significant differences were observed among the exploratory subgroups. Given the limited number of studies and statistical power, the analyses could not determine whether diabetes status or the inclusion of heart failure in the MACE definition accounted for the observed heterogeneity. Moreover, the secondary analysis of MACE components revealed that the association between a higher TyG index and coronary revascularization was not statistically significant. Given the limited number of studies underpinning this analysis, the lack of statistical significance in revascularization outcomes may reflect limited statistical power rather than a lack of evidence. More large-scale prospective studies are required that clarify the status of diabetes, employ standardized definitions of MACE, uniform follow-up intervals, and comparable multivariate adjustment strategies to assess the strength of the continuous association between the TyG index and postoperative outcomes, as well as its potential factors. Notably, the supplementary analysis was based on unadjusted cumulative event counts and did not account for covariate adjustment or variations in follow-up duration. Therefore, it is considered only as supporting evidence.

Although elevated TyG levels are associated with an increased risk of postoperative MACE, existing evidence does not necessarily confer robust prognostic discriminative ability at the individual level. In the two studies reporting discriminatory indices, the AUC values were 0.593 and 0.624, respectively, with sensitivity ranging from 0.509 to 0.671 and specificity from 0.681 to 0.689. These findings suggest that the TyG index alone is insufficient to accurately distinguish which patients will develop postoperative MACE. The findings regarding incremental prognostic value also remain inconsistent. Wu et al. reported an increased C-statistic, along with positive estimates for the NRI and IDI, whereas Wang et al. observed a slight decrease in the C-statistic. The NRI and IDI estimates were also positive. These findings did not consistently demonstrate improved overall prognostic discrimination after adding TyG. Given the small number of independent study cohorts and varying reference models, the findings remain exploratory. Prospective studies should use standardized models to externally validate the incremental value of TyG and document variations in discriminatory power, calibration, and clinical net benefit.

Drug exposure and metabolism management could impact TyG measurements and postoperative outcomes. Statins lower triglyceride levels and cardiovascular risk, yet may moderately elevate blood glucose, implying their net effect on TyG varies with therapeutic intensity and adherence (30). Antidiabetic therapy directly affects the fasting blood glucose component of TyG, and while sodium–glucose cotransporter 2 (SGLT2) inhibitors and glucagon-like peptide-1 (GLP-1) receptor agonists mitigate cardiovascular events beyond what baseline blood glucose levels alone could capture (31–34). Insulin decreases blood glucose levels, yet it may also identify patients with longer-standing or more severe diabetes, thereby generating potential confounding factors. Different studies have not consistently reported data on drug type, dosage, duration, and adherence. Although the included studies explicitly specified fasting triglyceride and blood glucose measurements, there was inadequate reporting of fasting duration and blood sampling times relative to the timing of drug administration and surgery, introducing measurement variability. Perioperative blood glucose control could independently impact postoperative complications and survival (35). Current guidelines for cardiac surgery emphasize active perioperative blood glucose monitoring and insulin therapy when necessary, while acknowledging that the optimal blood glucose target remains unclear (36). Therefore, differences in perioperative blood glucose management strategies may have contributed to variations in subsequent outcomes and the observed strength of the associations. Further studies should standardize fasting and sampling conditions and record statin intensity, specific antidiabetic medications, insulin usage, glycated hemoglobin (HbA1c), and longitudinal perioperative glucose exposure.

### 4.2 Limitations

Only six retrospective cohort reports, representing four independent cohort families involving 5,978 participants, were conducted in China. The quantitative analysis involved only two to four distinct cohorts, which restricted the precision of pooled estimates, heterogeneity assessment, subgroup comparisons, and the evaluation of publication bias. Furthermore, differences among studies in terms of study population selection, MACE definitions, follow-up duration, and strategies for covariate adjustment exacerbate heterogeneity and affect the comparability of the pooled effect sizes. In addition, information regarding drug exposure, fasting status, timing of sampling, and perioperative blood glucose management has not been adequately reported.

## 5 Conclusion

Among patients undergoing CABG, a higher TyG index was associated with an increased risk of subsequent MACE in both adjusted time-to-event and supportive event-based analyses. Associations were also observed with all-cause mortality, nonfatal stroke, and nonfatal myocardial infarction, whereas the association with revascularization was not statistically significant. Nevertheless, the TyG index has limited independent discriminatory power. Therefore, while the TyG index may serve as an accessible supplementary metabolic risk marker, prospective validation in geographically diverse populations is required before it is routinely used for individual risk prediction.

## Data Availability

The original contributions presented in the study are included in the article/**Supplementary Material**. Further inquiries can be directed to the corresponding author.
