# Supplementary material for: Triglyceride–glucose index as a metabolic risk marker for adverse outcomes after coronary artery bypass grafting: a systematic review and meta-analysis
Source: Front Endocrinol (Lausanne). 2026 Sep 11;17:1909829. doi: 10.3389/fendo.2026.1909829 (PMC13612311; doi:10.3389/fendo.2026.1909829)
Supplement: Supplementary file 1 [file SupplementaryFile1.docx]

**Additional file 1**

**Appendix 1.** Detailed characteristics of the included studies.

| Author  (Year) | Patient population | Participating center(s) | Enrollment period | Inclusion and exclusion criteria | Outcome definition | Potential cohort overlap |
| --- | --- | --- | --- | --- | --- | --- |
| Chen 2022 | 1,578 adults with T2DM undergoing isolated OPCABG | Beijing Anzhen Hospital, Capital Medical University | Sep 2017-Jun 2019 | Patients with T2DM undergoing isolated OPCABG. Key exclusions included concomitant or redo surgery, perioperative death, cardiogenic shock, BMI >45 kg/m², suspected familial hypertriglyceridemia, and missing follow-up. | MACCE: all-cause death, nonfatal MI, nonfatal stroke, or symptomatic graft failure. Symptomatic graft failure required typical post-discharge angina plus graft occlusion or ≥70% stenosis on CCTA. | No apparent overlap identified |
| Zhang 2022 | 386 adults with T2DM after isolated CABG | Nanjing Drum Tower Hospital | Jan 2014-Dec 2020 | Patients with T2DM undergoing isolated CABG. Key exclusions included acute MI, untreated new-onset diabetes, missing baseline data, and loss to follow-up. | MACE: all-cause death, nonfatal MI, nonfatal stroke, repeat revascularization (CABG or PCI), or rehospitalization for heart failure. | No apparent overlap identified |
| Wu 2023a | 1,013 adults undergoing first-time isolated CABG; mixed diabetes status | Qilu Hospital of Shandong University | Jun 2014-Dec 2017 | Consecutive patients undergoing isolated first-time CABG. Key exclusions included concomitant surgery, suspected familial hypertriglyceridemia, and missing data required for EuroSCORE II calculation. | MACE: all-cause death, repeat coronary revascularization, nonfatal MI, or nonfatal stroke. | Shandong CABG cohort family |
| Wu 2023b | 1,225 adults undergoing first-time isolated CABG; mixed diabetes status | Shandong Provincial Hospital; The Second Hospital of Shandong University; Qilu Hospital of Shandong University | Jun 2014-Jun 2018 | Consecutive CABG patients. Key exclusions included concomitant surgery, previous CABG, urate-lowering treatment, suspected familial hypertriglyceridemia, and missing SUA or TyG data. | MACE: first occurrence of all-cause death, nonfatal MI, nonfatal stroke, or repeat ischaemia-driven coronary revascularization; in-hospital death was excluded. | Shandong CABG cohort family |
| Sun 2025 | 1,472 adults undergoing CABG; mixed diabetes status | Shandong Provincial Hospital, The Second Hospital of Shandong University, and Qilu Hospital of Shandong University | Jun 2014-Jan 2019 | Adults aged ≥18 years undergoing CABG. Key exclusions included concomitant valve/congenital surgery, previous CABG, malignancy, and missing data required for the insulin-resistance indices; patients without completed follow-up were not analyzed. | MACE: all-cause death, nonfatal MI, repeat revascularization (PCI or CABG), or nonfatal stroke (ischaemic, haemorrhagic, or unclassified). | Shandong CABG cohort family* |
| Wang 2026 | 304 adults undergoing CABG; mixed diabetes status | The Affiliated Hospital of Qingdao University | Jan 2018-Dec 2022 | Adults undergoing CABG with preoperative CCTA, complete records, and follow-up. Key exclusions included concomitant surgery, previous PCI/CABG, severe cardiomyopathy, severe pulmonary/hepatic/renal disease, malignancy, TG ≥5.65 mmol/L, and missing key data. | MACE: all-cause death, repeat coronary revascularization, heart failure, severe arrhythmia, or stroke. | No apparent overlap identified |

Potential cohort overlap was assessed by comparing the participating centers, enrollment periods, patient populations, and eligibility criteria.

Wu 2023a, Wu 2023b, and Sun 2025 were considered reports from the same or substantially overlapping Shandong CABG cohort family based on overlapping participating centers, enrollment periods, patient populations, and authorship. Only one report from this cohort family was included in any given quantitative synthesis.

*Sun 2025 was selected as the representative report for the primary time-to-event synthesis because it included the largest, most comprehensive cohort and the broadest enrollment period.

**Appendix 2.** Outcomes extraction of included studies.

| Study | N | MACE (Higher vs. lower TyG index) | | | HR per 1 unit  increase (95% CI) | HR（95% CI）for  T3 vs. T1 | HR（95% CI）for  T2 vs. T1 | Prognostic / Predictive Index | | | | | |
| --- | --- | --- | --- | --- | --- | --- | --- | --- | --- | --- | --- | --- | --- |
|  |  | Total | Details | Categorical TyG exposure definition |  |  |  | AUC  (95% CI) | TP | FP | FN | TN | ROC-derived optimal TyG cut-off |
| Chen 2022 | 1578 | 88(526) vs. 88(1052) | All-cause death (6vs.11) Nonfatal MI (20vs.19) Nonfatal stroke (21vs.19) Symptomatic graft failure (40 vs.39) | Higher vs lower groups; mean (SD): 9.83 (0.34) vs 8.78 (0.41); threshold NR | 1.954(1.508-2.533) | 2.133(1.347-3.377) | 1.726 (1.093-2.725) | NR | NA | NA | NA | NA | NR |
| Zhang 2022 | 386 | 65(193) vs. 39(193) | All-cause death (25vs.13)  Nonfatal MI (9vs.3)  Non-fatal stroke (10vs.6) Rehospitalization for heart failure (46 vs.21)  Revascularization (CABG or PCI) (4vs.3) | Dichotomized at the median of 9.17 | 12.926 (3.457-48.323), not pooled* | NA | NA | NR | NA | NA | NA | NA | NR |
| Wu 2023a | 1013 | 98(337) vs. 113(676) | All-cause death (31vs.43)  Nonfatal MI (31vs.30) Nonfatal stroke (22vs.24) Coronary artery revascularisation (20vs.27)  Cardiac death (27vs.22) | Higher vs lower groups; mean (SD): 9.41 (0.45) vs 8.38 (0.37); threshold NR | 1.62 (1.27-2.07) | 2.34 (1.60-3.43) | 1.36 (0.92-1.99) | 0.607 (0.577-0.638) | 126 | 303 | 95 | 489 | 8.77 |
| Wu 2023b | 1225 | 145(451) vs. 118(774) | NR | ROC-derived cut-off: >8.87 vs ≤8.87 | 1.53 (1.24-1.89) | NA | NA | NR | 145 | 305 | 118 | 657 | 8.87 |
| Sun 2025 | 1472 | NA | NA | Tertiles; numerical boundaries NR | 1.488 (1.239-1.787) | 2.077 (1.549-2.784) | 1.180 (0.875-1.592) | AUC with TyG cut-off 8.9=  0.593（0.557-0.629） | 171 | 362 | 165 | 774 | 8.9 |
| Wang 2026 | 304 | 55(124) vs. 27(180) | NR | Dichotomized at 8.65 | 1.47 (1.05–2.29) | NA | NA | 0.624 (0.554–0.694) | 55 | 69 | 27 | 153 | NR |

*Zhang 2022 reported an adjusted HR of 12.926 (95% CI 3.457–48.323) for the TyG index without specifying the corresponding exposure increment. Therefore, this estimate was not treated as a per-unit HR and was excluded from the continuous meta-analysis. The median-based higher-versus-lower comparison was retained in the categorical meta-analysis.

For Wang et al. (2026), the published HR and 95% CI were 1.47 (1.05–2.29). Because the reported CI was asymmetric on the logarithmic scale, the published point estimate was retained, while the SE was derived from the CI limits. Consequently, the forest plot displays the corresponding Wald-based CI generated from the derived SE.

Categorical TyG exposure definition refers to the method used to define exposure groups for categorical analyses and should be distinguished from the ROC-derived optimal cut-off used for outcome discrimination. NA not applicable, NR not reported.

**Appendix 3.** Variables Included in the Most Fully Adjusted Models.

| Study | Adjustment Variables |
| --- | --- |
| Chen 2022 | Age, sex, BMI, current smoking, hypertension, previous MI, previous stroke, previous PCI, cardiac failure, CKD, preoperative LVEF, insulin dependence, LDL-C, HDL-C, clinical diagnosis, extent of CAD, left main disease, complete revascularization, and use of IABP. |
| Zhang 2022 | LVEF, NYHA functional class III/IV, triglycerides, fasting glucose, HDL-C, creatinine, BUN, off-pump CABG, and number of grafts. |
| Wu 2023a | TyG, EuroSCORE II, BMI, current smoking, current drinking, FH-CAD, DM, hypertension, hyperlipidemia, left main disease, multivessel disease, duration of surgery, OPCABG, number of grafts, use of arterial grafts, LDL-C, HDL-C, antiplatelet drugs, statins, and hypoglycemic drugs. |
| Wu 2023b | TyG, SUA, age, LVEF, left main disease, current smoking, DM, hypertension, TC, LDL-C, eGFR, sex, BMI, current drinking, hyperlipidemia, duration of surgery, OPCABG, number of grafts, use of arterial grafts, antiplatelet drugs, statins, hypoglycemic drugs, and urate-lowering drugs. |
| Sun 2025 | Age, sex, LVEF, current smoking, hypertension, DM, left main disease, duration of surgery, number of grafts, eGFR, UA, LDL-C, current drinking, previous MI, previous stroke, FH-CAD, OPCABG, use of arterial grafts, and antiplatelet drugs. |
| Wang 2026 | TyG, BMI, LVEF, age, sex, left main disease, triglyceride level, and serum creatinine. |

BMI body mass index, BUN blood urea nitrogen, CABG coronary artery bypass grafting, CAD coronary artery disease, CKD chronic kidney disease, DM diabetes mellitus, eGFR estimated glomerular filtration rate, EuroSCORE II European System for Cardiac Operative Risk Evaluation II, FH-CAD family history of coronary artery disease, HDL-C high-density lipoprotein cholesterol, IABP intra-aortic balloon pump, LDL-C low-density lipoprotein cholesterol, LVEF left ventricular ejection fraction, MI myocardial infarction, NYHA New York Heart Association, OPCABG off-pump coronary artery bypass grafting, PCI percutaneous coronary intervention, SUA serum uric acid, TC total cholesterol, TyG triglyceride-glucose, UA uric acid.

**Appendix 4.** Risk of bias assessments according to the Newcastle-Ottawa Scale.

**Appendix Table 4A.** Newcastle–Ottawa Scale scores of the included studies.

| Study | Selection Comparability Outcome | | | | | | | | | Total |
| --- | --- | --- | --- | --- | --- | --- | --- | --- | --- | --- |
|  | S1 | S2 | S3 | S4 | C1 | C2 | O1 | O2 | O3 |  |
| Chen 2022 | / | ★ | ★ | / | ★ | ★ | ★ | ★ | / | 6 |
| Zhang 2022 | / | ★ | ★ | / | ★ | / | ★ | ★ | / | 5 |
| Wu 2023a | ★ | ★ | ★ | / | ★ | ★ | ★ | ★ | / | 7 |
| Wu 2023b | ★ | ★ | ★ | / | ★ | ★ | ★ | ★ | / | 7 |
| Sun 2025 | ★ | ★ | ★ | / | / | ★ | ★ | ★ | / | 6 |
| Wang 2026 | ★ | ★ | ★ | / | ★ | ★ | ★ | ★ | / | 7 |

S selection, C comparability, O outcome, CABG coronary artery bypass grafting. A filled star (★) indicates that the corresponding criterion was satisfied, whereas a slash (/) indicates that no star was awarded. The NOS for cohort studies comprises eight items and permits a maximum of nine stars, as the comparability domain can receive up to two stars.

**Appendix Table 4B.** Newcastle–Ottawa Scale items and study-specific scoring criteria.

CABG coronary artery bypass grafting, NOS, Newcastle–Ottawa Scale.

| Code | NOS item | Assessment options and scoring criteria |
| --- | --- | --- |
| S1 | Representativeness of the exposed cohort | a. Truly representative of patients undergoing CABG in the source population, such as a consecutive or broadly recruited cohort (★); b. Somewhat representative of patients undergoing CABG in the source population (★); c. Selected subgroup of patients; d. No description of cohort derivation. |
| S2 | Selection of the non-exposed cohort | a. The comparison cohort was drawn from the same source population as the exposed cohort (★); b. The comparison cohort was drawn from a different source; c. No description of the comparison cohort. |
| S3 | Ascertainment of exposure | a. Secure medical, laboratory, or electronic health records (★); b. Structured interview (★); c. Written self-report; d. No description. |
| S4 | Demonstration that the outcome of interest was not present at the start of the study | a. The postoperative outcome of interest had not occurred at the beginning of follow-up (★); b. The outcome was already present, or this could not be determined. |
| C1 | Comparability for the most important confounding factor | The adjusted analysis controlled for age, which was considered the most important prespecified confounding factor (★). |
| C2 | Comparability for additional confounding factors | The adjusted analysis controlled for at least one additional relevant cardiovascular, metabolic, or surgical factor, such as sex, diabetes mellitus, hypertension, smoking, body mass index, lipid levels, renal function, left ventricular function, or operative characteristics (★). |
| O1 | Assessment of outcome | a. Independent or blinded outcome assessment (★); b. Medical-record or registry linkage (★); c. Patient self-report alone; d. No description. |
| O2 | Was follow-up sufficiently long for outcomes to occur? | a. Yes, the follow-up duration was at least 12 months, which was considered sufficient for postoperative cardiovascular events to occur (★); b. No, the follow-up duration was shorter than 12 months or was not reported. |
| O3 | Adequacy of cohort follow-up | a. Complete follow-up, with all participants accounted for (★); b. Follow-up rate ≥80%, or losses to follow-up were adequately described and unlikely to introduce substantial bias (★); c. Follow-up rate <80% without adequate description of losses; d. No statement regarding completeness of follow-up. |

**Appendix 5.** Leave-one-out sensitivity analysis of the pooled HRs for MACE per 1-unit increase in the TyG index.

**Appendix 6.** Forest plots of the association between TyG index tertiles and MACE.

(A) Highest versus lowest TyG tertile (T3 vs T1). (B) Middle versus lowest TyG tertile (T2 vs T1). Pooled HRs and 95% CIs were calculated using random-effects models. CI confidence interval, HR hazard ratio, MACE major adverse cardiovascular events, T tertile, TyG triglyceride-glucose index.

**Appendix 7.** Leave-one-out sensitivity analysis of the pooled OR for high vs. low TyG index and MACE.

**Appendix 8.** Exploratory subgroup analyses for the TyG index and the risk of MACE.

| Subgroup | Number of Included Studies | Heterogeneity | | Meta-analysis | | *P* for subgroup difference |
| --- | --- | --- | --- | --- | --- | --- |
|  |  | *P*-value | I^2^ | OR（95% CI） | *P*-value |  |
| Overall analysis | 4 | 0.102 | 52% | 2.585 (1.967, 3.397) | ＜0.001 |  |
| Diabetes status |  |  |  |  |  | 0.149 |
| T2DM population | 2 | 0.744 | 0% | 2.136 (1.646, 2.773) | ＜0.001 |  |
| General CABG population | 2 | 0.082 | 67% | 3.275 (1.950, 5.500) | ＜0.001 |  |
| MACE definition |  |  |  |  |  | 0.636 |
| Not including heart failure | 2 | 0.403 | 0% | 2.436 (1.976, 3.002) | ＜0.001 |  |
| Including heart failure | 2 | 0.025 | 80% | 2.971 (1.341, 6.582) | 0.007 |  |

OR, odds ratio; CI, confidence interval; T2DM, type 2 diabetes mellitus; CABG, coronary artery bypass grafting; MACE, major adverse cardiovascular events. All estimates were calculated using random-effects models. Subgroup analyses were exploratory because each subgroup contained only two studies.

**Appendix 9.** Forest plot of the definition-based sensitivity analysis for the association between high TyG index and MACE.
