# Supplementary material for: Triglyceride–glucose index as a metabolic risk marker for adverse outcomes after coronary artery bypass grafting: a systematic review and meta-analysis
Source: Front Endocrinol (Lausanne). 2026 Sep 11;17:1909829. doi: 10.3389/fendo.2026.1909829 (PMC13612311; doi:10.3389/fendo.2026.1909829)
Supplement: Supplementary file 2 [file SupplementaryFile2.docx]

**Additional file 2**

**Table S1.** Search strategy.

| PubMed | | |
| --- | --- | --- |
| #1 | (triglyceride-glucose index) OR (triglyceride glucose index) OR (TyG index) OR (triglyceride and glucose index) | 21488 |
| #2 | Coronary Artery Bypass[MeSH Terms] | 58197 |
| #3 | Coronary Artery Bypass, Off-Pump[MeSH Terms]­ | 3811 |
| #4 | Internal Mammary-Coronary Artery Anastomosis[MeSH Terms] | 2445 |
| #5 | (Bypass, Coronary Artery) OR (Bypass Surgery, Coronary Artery) OR (Coronary Artery Bypass, Off Pump) OR (Internal Mammary Coronary Artery Anastomosis) OR (Coronary Artery Bypass) OR (Artery Bypass, Coronary) OR (Bypass, Aortocoronary) OR (Artery Bypasses, Coronary) OR (Bypasses, Coronary Artery) OR (Off Pump Coronary Artery Bypass) OR (Coronary Artery Bypass Surgery) OR (Coronary Artery Bypasses) OR (Off-Pump Coronary Artery Bypass) OR (Bypasses, Aortocoronary) OR (Beating Heart Coronary Artery Bypass) OR (Anastomosis, Internal Mammary Coronary Artery) OR (Aortocoronary Bypasses) OR (Coronary Artery Bypass, Beating Heart) OR (Anastomosis, Internal Mammary-Coronary Artery) OR (Coronary Artery Bypass Grafting) OR (Coronary Internal Mammary Artery Anastomosis) OR (Aortocoronary Bypass) OR (Coronary-Internal Mammary Artery Anastomosis) | 71602 |
| #6 | #2 OR #3 OR #4 OR #5 | 83413 |
| #7 | #1 AND #6 | 36 |
| Web of Science | | |
| #1 | **((TS=(triglyceride-glucose index)) OR TS=(triglyceride glucose index)) OR TS=(TyG index)** | 38317 |
| #2 | **((TS=(Coronary Artery Bypass)) OR TS=(Coronary Artery Bypass, Off-Pump)) OR TS=(Internal Mammary-Coronary Artery Anastomosis)** | 111563 |
| #3 | TS=("Coronary Internal Mammary Artery Anastomosis" OR  "Coronary Artery Bypass Grafting" OR  "Off-Pump Coronary Artery Bypass" OR  "Coronary Artery Bypass, Off-Pump" OR  "Artery Bypass, Coronary" OR  "Artery Bypasses, Coronary" OR  "Coronary Artery Bypass Surgery" OR  "Aortocoronary Bypass" OR  "Coronary-Internal Mammary Artery Anastomosis" OR  "Anastomosis, Internal Mammary Coronary Artery" OR  "Internal Mammary Coronary Artery Anastomosis" OR  "Aortocoronary Bypasses" OR  "Bypasses, Aortocoronary" OR  "Off-Pump Coronary Artery Bypass" OR  "Coronary Artery Bypasses" OR  "Bypass Surgery, Coronary Artery" OR  "Coronary Artery Bypass, Beating Heart" OR  "Beating Heart Coronary Artery Bypass") | 57322 |
| #4 | #2 OR #3 | 112434 |
| #5 | #1 AND #4 | 45 |
| EMBASE | | |
| #1 | 'triglyceride-glucose index':ti,ab,kw | 2102 |
| #2 | 'triglyceride glucose index':ti,ab,kw | 2096 |
| #3 | 'TyG index':ti,ab,kw | 2295 |
| #4 | #1 OR #2 OR #3 | 2850 |
| #5 | 'Coronary Artery Bypass, Off-Pump'/exp | 7277 |
| #6 | 'Coronary Artery Bypass'/exp | 97794 |
| #7 | 'Internal Mammary-Coronary Artery Anastomosis'/exp | 41910 |
| #8 | 'Coronary Internal Mammary Artery Anastomosis':ti,ab,kw OR 'Coronary Artery Bypass Grafting':ti,ab,kw OR 'Off Pump Coronary Artery Bypass':ti,ab,kw OR 'Coronary Artery Bypass, Off Pump':ti,ab,kw OR 'Bypasses, Coronary Artery':ti,ab,kw OR 'Artery Bypass, Coronary':ti,ab,kw OR 'Artery Bypasses, Coronary':ti,ab,kw OR 'Coronary Artery Bypass Surgery':ti,ab,kw OR 'Bypass, Aortocoronary':ti,ab,kw OR 'Coronary-Internal Mammary Artery Anastomosis':ti,ab,kw OR 'Bypass, Coronary Artery':ti,ab,kw OR 'Anastomosis, Internal Mammary Coronary Artery':ti,ab,kw OR 'Internal Mammary Coronary Artery Anastomosis':ti,ab,kw OR 'Aortocoronary Bypasses':ti,ab,kw OR 'Anastomosis, Internal Mammary-Coronary Artery':ti,ab,kw OR 'Bypasses, Aortocoronary':ti,ab,kw OR 'Off-Pump Coronary Artery Bypass':ti,ab,kw OR 'Aortocoronary Bypass':ti,ab,kw OR 'Coronary Artery Bypass':ti,ab,kw OR 'Coronary Artery Bypasses':ti,ab,kw OR 'Bypass Surgery, Coronary Artery':ti,ab,kw OR 'Coronary Artery Bypass, Beating Heart':ti,ab,kw OR 'Beating Heart Coronary Artery Bypass':ti,ab,kw | 73124 |
| #9 | #5 OR #6 OR #7 OR #8 | 149461 |
| #10 | #4 AND #9 | 16 |
| Cochrane Library | | |
| #1 | MeSH descriptor: [Coronary Artery Bypass] explode all trees | 6777 |
| #2 | MeSH descriptor: [Coronary Artery Bypass, Off-Pump] explode all trees | 543 |
| #3 | MeSH descriptor: [Internal Mammary-Coronary Artery Anastomosis] explode all trees | 107 |
| #4 | (Bypass, Coronary Artery):ti,ab,kw OR (Bypass Surgery, Coronary Artery):ti,ab,kw OR (Coronary Artery Bypass, Off Pump):ti,ab,kw OR (Internal Mammary Coronary Artery Anastomosis):ti,ab,kw OR (Coronary Artery Bypass):ti,ab,kw OR (Artery Bypass, Coronary):ti,ab,kw OR (Bypass, Aortocoronary):ti,ab,kw OR (Artery Bypasses, Coronary):ti,ab,kw OR (Bypasses, Coronary Artery):ti,ab,kw OR (Off Pump Coronary Artery Bypass):ti,ab,kw OR (Coronary Artery Bypass Surgery):ti,ab,kw OR (Coronary Artery Bypasses):ti,ab,kw OR (Off-Pump Coronary Artery Bypass):ti,ab,kw OR (Bypasses, Aortocoronary):ti,ab,kw OR (Beating Heart Coronary Artery Bypass):ti,ab,kw OR (Anastomosis, Internal Mammary Coronary Artery):ti,ab,kw OR (Aortocoronary Bypasses):ti,ab,kw OR (Coronary Artery Bypass, Beating Heart):ti,ab,kw OR (Anastomosis, Internal Mammary-Coronary Artery):ti,ab,kw OR (Coronary Artery Bypass Grafting):ti,ab,kw OR (Coronary Internal Mammary Artery Anastomosis):ti,ab,kw OR (Aortocoronary Bypass):ti,ab,kw OR (Coronary-Internal Mammary Artery Anastomosis):ti,ab,kw | 14992 |
| #5 | #1 OR #2 OR #3 OR #4 | 15023 |
| #6 | (triglyceride-glucose index):ti,ab,kw OR (triglyceride glucose index):ti,ab,kw OR (TyG index):ti,ab,kw | 189 |
| #7 | #5 AND #6 | 10 |
